# Study of fluoroquinolones resistance in rifampicin‐resistant tuberculosis patients in Beijing: Characteristics, trends, and treatment outcomes

**DOI:** 10.1002/ame2.12505

**Published:** 2025-02-05

**Authors:** Wang Nenhan, Tian Lili, Zhao Yanfeng, Chen Shuangshuang, Tao LiYing, Li Qiao, Li Chuanyou, Dai Xiaowei

**Affiliations:** ^1^ Beijing Center for Disease Prevention and Control Beijing People's Republic of China; ^2^ Institute of Pathogen Biology Chinese Academy of Medical Sciences Beijing People's Republic of China

**Keywords:** drug resistance, levofloxacin, moxifloxacin, *mycobacterium tuberculosis*, pre‐XDR‐TB

## Abstract

**Background:**

China is a high‐burden country for multidrug‐resistant tuberculosis/rifampin‐resistant tuberculosis (MDR/RR‐TB). Fluoroquinolones (FQs) are key drugs for the treatment of patients with MDR/RR‐TB. However, research on the resistance of FQs in Beijing is limited.

**Methods:**

We collected clinical isolates from all patients with pulmonary TB in Beijing from January 2016 to December 2021, conducted drug‐sensitivity tests and sequencing for levofloxacin (LFX) and moxifloxacin (MFX), and collected the treatment plans and outcomes of the patients.

**Results:**

A total of 8512 clinical isolates were collected from patients with pulmonary TB, and 261 RR‐TB strains were screened. The proportions of drug‐sensitive and drug‐resistant strains significantly differed by age group and treatment history. The rates of LFX and MFX resistance were 27.6% (72/261) and 36.4% (95/261), respectively. The detection rates of MDR‐TB and pre‐extensively drug‐resistant TB (pre‐XDR‐TB) were 73.2% (191/261) and 36.4% (95/261), respectively, and the trends were significant (*χ* ^2^ trend = 9.995, *p* = 0.002; *χ* ^2^ trend = 12.744, *p* = 0.026). Among the 261 RR‐TB strains, 14.9% (24/261) were sensitive to LFX but resistant to MFX. Among the four patients with LFX‐resistant TB who received LFX treatment failed in three patients(Fisher's exact test, *p* = 0.009). The common mutation sites were 94 and 90 in gyrA. A novel mutation Ala90Ser was discovered.

**Conclusions:**

FQs resistance trends in RR‐TB patients in Beijing are striking. Strains showed incomplete cross‐resistance to LFX and MFX. Testing for FQs resistance and developing a reasonable treatment plan are recommended. Attention should be given to the changing trends in MDR‐TB and pre‐XDR‐TB.

## Graphical abstract

We collected the clinical isolates from all patients with pulmonary TB in Beijing from January 2016 to December 2021, A total of 8,512 clinical isolates were collected, and 261 RR‐TB strains were screened. We conducted the drug sensitivity tests and sequencing for LFX and MFX. The MICs of the strains were mainly distributed at 0.25 μg/mL for both drugs, and among the 261 strains of RR‐TB, 27.2% were resistant to both LFX and MFX, 0.4% were resistant to LFX but sensitive to MFX, 9.2% were sensitive to LFX but resistant to MFX, and 63.2% were sensitive to both LFX and MFX. The MDR‐TB detection rate over this period significantly declined after rebounding in 2019 and 2020, and the pre‐XDR‐TB detection rate slowly decreased from 2016 to 2018 and then continued to decline after a significant increase in 2019. By 2021, the rate had recovered to the 2018 level. After sequencing, we found that the common mutation sites were Asp94, Ala90, Asp91, and Asp88.

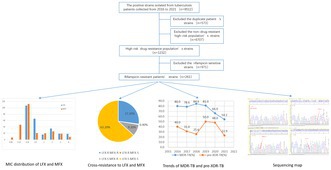

## 1 INTRODUCTION

Drug‐resistant tuberculosis (DR‐TB) is characterized by long transmission time, complex treatment scheme, long treatment term, high treatment cost, low treatment compliance, and low cure rate. Therefore, the preventing and controlling  of DR‐TB globally presents a severe challenge. China is a high‐burden country (HBC) for multidrug‐resistant /rifampin‐resistant (MDR/RR‐TB). In 2022, there were approximately 748 000 cases of TB in China, including approximately 30 000 cases of MDR/RR‐TB, ranking third among HBCs after India and Indonesia.^1^ The situation of TB prevention and control in China remains grim.

The “WHO consolidated guidelines on tuberculosis: Module 4: Treatment: Drug‐Resistant tuberculosis treatment, 2020” states that long‐term regimens for patients with MDR/RR‐TB should include levofloxacin (LFX) and moxifloxacin (MFX), and all patients with MDR/RR‐TB should undergo drug resistance tests (DSTs) for fluoroquinolones (FQs) before starting treatment.^2^ The rapid identification of FQs resistance and adjustment of treatment plans based on DST results can facilitate accurate treatment and reduce TB transmission and mortality. Although traditional DSTs (both on solid medium and in liquid culture) are the gold standards for clinical DST, they have several drawbacks, such as being time‐consuming, laborious, and complex.^3^ To quickly obtain phenotypic DST results, microplate‐based DST reagents have been continuously introduced in recent years. These tests use a 96‐well microplate to simultaneously detect multiple drugs, and each drug can be tested at multiple concentrations. In addition to classifying isolates as susceptible or resistant to various drugs, the minimum inhibitory concentration (MIC) can be obtained. Therefore, we can understand the impact of drug resistance levels on therapeutic efficacy.^4^ As core drugs for the treatment of MDR/RR‐TB, LFX and MFX play important roles in the treatment of DR‐TB. However, Beijing, an international metropolis with a resident population of 21.5 million, lacks data on FQs resistance among patients with RR‐TB.^5^

This study applied microplate‐based DST and sequencing for LFX and MFX, and drug resistance, the degree of resistance, and trends of FQs resistance among *Mycobacterium tuberculosis* (MTB) isolates from patients with RR‐TB in Beijing were examined. The cross‐resistance of strains to LFX and MFX and the outcomes of treatment were also analyzed. The research results are crucial for the clinical treatment of RR‐TB in Beijing and the development of prevention and control strategies.

## 2 METHODS

### 2.1 Research object

This retrospective study encompassed MTB clinical isolates from patients who were treated in TB prevention and control institutions and designated hospitals in Beijing from January 2016 to December 2021. All strains were submitted to traditional DSTs for rifampicin and isoniazid. Strains that were resistant to rifampicin were submitted to DSTs and sequencing for LFX and MFX.

### 2.2 Subculture of MTB clinical isolates

The MTB clinical isolates were removed from a −80°C refrigerator and restored to room temperature. Then, 100 μL of the bacterial liquid was transferred to neutral Löwenstein–Jensen medium(Celnovte Biotechnology Co, Zhengzhou, Henan). The culture medium was rotated slowly to evenly spread the bacterial solution throughout the inclined surface. The culture tubes were placed in a 37°C incubator (Memmert IPP260, Schwabach, Germany) for cultivation, and colony growth was observed weekly.

### 2.3 Proportional DST

All strains underwent DSTs using the proportional method,^6^ and the drug concentration was 40 μg/mL for rifampin and 0.20 μg/mL for isoniazid. The control medium was neutral Löwenstein–Jensen medium. The drug resistance rate was calculated as follows: number of colonies growing on drug‐containing medium/number of colonies growing on control medium × 100%. Drug resistance was indicated by a drug resistance rate of ≥1%.

### 2.4 Microplate DST

We used the Tuberculosis Mycobacterium (TB) Drug Susceptibility MIC Plate suit (BASO Diagnostics Inc., Zhuhai, China) to perform the DST. Several colonies were selected during the logarithmic growth period (recovered within 1 month) using a sterile loop. A 1 McFarland standard equivalent was prepared using an Ultrasonic Milling Instrument (BACspreade1100C, TB Healthcare). Suspensions were diluted 100‐fold by adding 50 μL of the 1 McFarland suspension to 5 mL of diluent (7H9 + 10% Oleic Acid, Albumin, Dextrose, and Catalase medium (OADC)). Aliquots of 100 μL of the inoculum were distributed to each well using an auto‐inoculator (BSJ‐9612, BASO Diagnostics Inc., Zhuhai, China). Negative control wells (containing 100 μL of the diluent without drugs) and positive control wells (containing 100 μL of the inoculum without drugs) were created. After the samples were added, plates were sealed and incubated at 37°C. After 10–14 days, the results were interpreted using a biological drug sensitivity analysis program (BSP‐TB96, BASO Diagnostics Inc.). The concentration ranges (breakpoints) in the experiment were 0.25–8 μg/mL (1 μg/mL) for LFX^4^ and 0.06–8 μg/mL (0.25 μg/mL) for MFX.^7^

### 2.5 Interpretation of the microplate DST results

MTB H37Rv (ATCC 27294) was used for quality control for each batch. When the DST result of MTB H37Rv was within the quality control range (with bacterial growth in the positive control well and no bacterial growth in the negative control well), the batch of experiments was deemed successful. Repeated experiments were required if there was no colony growth or contamination in the positive control wells. The MIC was defined as the lowest concentration without considerable visible bacterial growth compared to the positive controls.

### 2.6 DNA extraction

Colonies were scraped in logarithmic growth phase and were placed in Eppendorf centrifuge tubes containing 1 mL of normal saline. Then they were made inactive at 99°C for 10 min and centrifuged at 120 00×*g* for 2 min. The supernatant was taken as the gene amplification template.

### 2.7 Amplification and sequencing of fluoroquinolone resistance genes

The lengths of the gyrA and gyrB gene fragments of the FQs resistance gene were 314 bp and 269 bp, respectively. Forward primer sequence of gyrA gene: gyrA‐F‐5 “‐ GGGTGCTATGCCAATGTGTGTCG‐3,” reverse primer sequence of gyrA gene: gyrA‐R‐5 “‐ GCCGTAGTTAGGATGA‐3,” forward primer sequence of gyrB gene: gyrB‐F‐5 “‐ TTCGATGTCCAGGCGGATACTTCC‐3,” reverse primer sequence of gyrB gene: gyrB‐R‐5 “‐ ACACATGCCCGTTCGATGAGCC‐3.” The amplification system was as follows: 25 μL of 2 × Taq Mix, 1 μL of forward primer, 1 μL of reverse primer, 1 μL of genomic DNA, and the remaining volume was filled with ddH_2_O to a total volume of 50 μL. The gene amplification conditions were as follows: pre‐denaturation at 94°C for 5 min; 94°C for 15 s, 60°C for 10 s, 72°C for 5 min, 34 cycles; extend at 72°C for 5 min. The gene amplification products were subjected to electrophoresis detection and sent to Gene‐Optimal Science & Technology Co., Ltd. (Shanghai, China) for sequencing. The sequencing results were compared and analyzed using the standard sensitive strain H37Rv gene sequence using BioEdit software.

### 2.8 Statistical analysis

Statistical analysis of the data was conducted using SPSS 19.0(IBM, Armonk, NY, USA). Numerical data were presented as the rate or composition ratio. Intra‐group differences were compared using the chi‐squared test. The chi‐square trend test was used to determine changes in detection rates, and *p* < 0.05 was considered statistically significant (the corrected chi‐square value was calculated when the theoretical frequency of the four grid table was 1 < *T* < 5, and Fisher's exact test was used when *T* < 1).

### 2.9 Definitions

RR‐TB: infection with an MTB strain with rifampin resistance.

MDR‐TB: infection with an MTB strain resistant to both rifampicin and isoniazid.

Pre‐XDR‐TB: infection with an MDR/RR‐TB strain that is additionally resistant to any FQs.^8^

Cross‐resistance: MTB is resistant to one anti‐TB drug while also developing resistance to another anti‐TB drug.

Bidirectional cross‐resistance: MTB is simultaneously resistant to two drugs. It can be divided into two types: complete and incomplete.^9^

Treatment Outcome: According to the “Guidelines for Chemotherapy of Drug resistant tuberculosis (2019)”,^9^ the treatment outcomes of RR‐TB are classified as cure, completion of treatment, failure, death, loss to follow‐up, and failure to evaluate. Treatment success includes cure and completion of treatment.

High‐risk population for drug resistance^10^: Mainly includes patients with recurrent treatment failure/chronic bacterial discharge, pathogen‐positive patients with close contact with patients with drug‐resistant TB, patients who failed initial treatment, patients with recurrence or relapse, and patients who remain positive for sputum smear or culture during the treatment process.

## 3 RESULTS

### 3.1 Population

**FIGURE 1:**
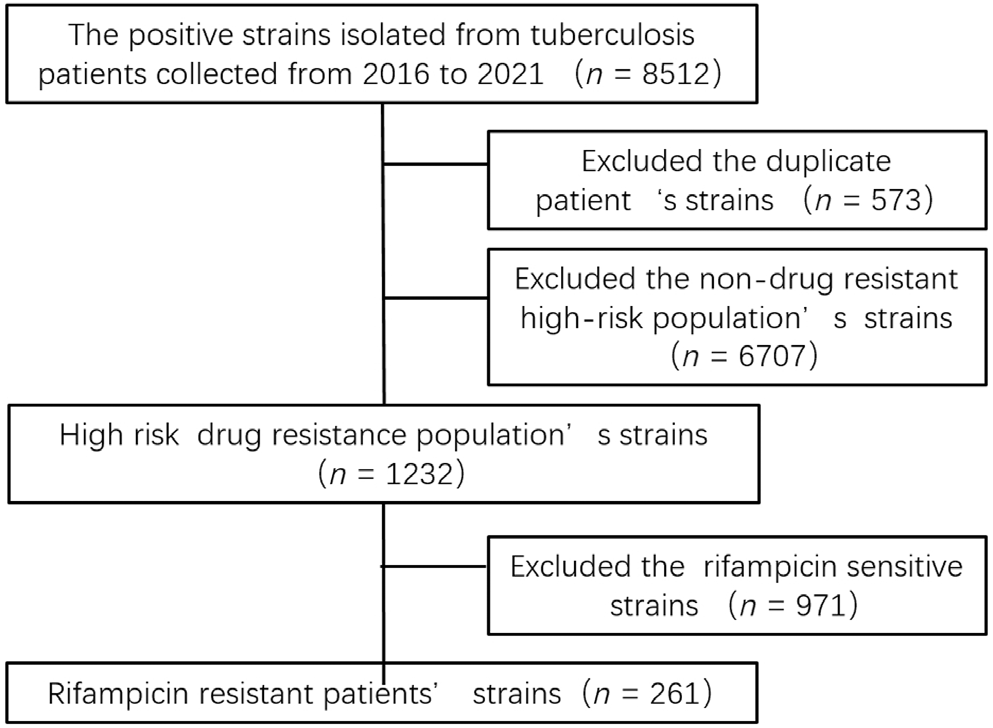
Loading and discharging process diagram.

We collected 8512 MTB clinical isolates, and after 573 duplicate strains were removed, 1232 strains from the high‐risk drug‐resistant population were subjected to the proportional DST, which identified 261 RR strains. The specific process is presented in Figure 1.

### 3.2 General situation of patients with RR‐TB

The 261 patients with RR‐TB included 187 men and 74 women with a median age (quartile) of 41 years (29, 55). The majority of patients were aged 20–59 years. The number of newly treated patients was higher than that of retreated patients. Most patients resided in Beijing. The number of cases was highest in central urban areas (Dongcheng, Xicheng, Haidian, Chaoyang, Shijingshan, Fengtai), followed by suburban areas (Tongzhou, Daxing, Changping, Fangshan, Shunyi, Mentougou), whereas the number of cases was lowest in remote suburban areas (Miyun, Pinggu, Huairou, Yanqing). The proportions of patients with resistance to LFX and MFX significantly differed by age and treatment history, whereas no differences were observed related to sex, registered residence, and patient origin (Table 1).

**TABLE 1:** General situation of patients with RR‐TB and comparison of intra‐group differences.

	RR‐TB (*n* = 261)	LFX				MFX			
		Drug resistance (*n* = 72)	Drug sensitivity (*n* = 189)	*χ* ^2^	*p*	Drug resistance (*n* = 95)	Drug sensitivity (*n* = 166)	*χ* ^2^	*p*
Sex									
Man	187 (71.6)	47 (65.3)	140 (74.1)	1.986	0.159	63 (66.3)	124 (74.7)	2.090	0.148
Woman	74 (28.4)	25 (34.7)	49 (25.9)			32 (33.7)	42 (25.3)		
Age(years)									
<20	11 (4.2)	0	11 (5.8)	24.181	0.001	1 (1.1)	10 (6.0)	24.051	0.001
20–59	199 (76.2)	45 (62.5)	154 (81.5)			61 (64.2)	138 (83.1)		
≥60	51 (19.6)	27 (37.5)	24 (12.7)			33 (34.7)	18 (10.8)		
Registered residence									
Beijing	151 (57.9)	47 (65.3)	104 (55.0)	2.247	0.134	59 (62.1)	92 (55.4)	1.107	0.293
Other places	110 42.1)	25 (34.7)	85 (45.0)			36 (37.9)	74 (44.6)		
Treatment history									
New Case	191 (73.2)	43 (59.7)	148 (78.3)	9.175	0.002	62 (65.3)	129 (77.7)	4.770	0.029
Retreat	70 (26.8)	29 (40.3)	41 (21.7)			33 (34.7)	37 (22.3)		
Address									
Downtown	129 (49.4)	38 (52.8)	91 (48.1)	0.647	0.724	49 (51.6)	80 (48.2)	0.422	0.810
Outskirts	89 (34.1)	24 (33.3)	65 (34.4)			32 (33.7)	57 (34.3)		
Outer suburbs	43 (16.5)	10 (13.9)	33 (17.5)			14 (14.7)	29 (17.5)		

Abbreviations: LFX, levofloxacin; MFX, moxifloxacin.

### 3.3 Microplate DST results

**FIGURE 2:**
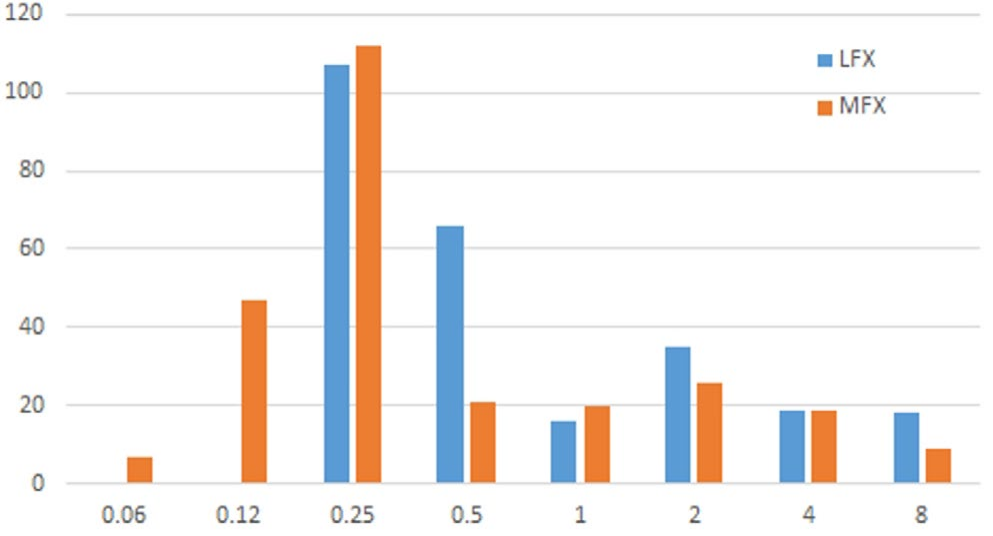
Minimum inhibitory concentration (MIC) distribution of levofloxacin (LFX) and moxifloxacin (MFX).

We performed LFX and MFX DSTs for the 261 strains from patients with RR‐TB using the microplate method. As 1 μg/mL was the critical concentration for low‐concentration drug resistance, the LFX resistance rate was 27.6% (72/261). As 0.25 μg/mL was the critical concentration for low‐concentration drug resistance, the MFX resistance rate was 36.4% (95/261). The MICs of the strains were mainly distributed at 0.25 μg/mL for both drugs (Figure 2; Table 2).

**TABLE 2:** Microplate DST results.

Drug	MIC distribution of rifampicin‐resistant *Mycobacterium tuberculosis*								Concentration range (μg/mL)	Cutoff value (μg/mL)	Resistance rate in RR‐TB (%)
	≤0.06	0.12	≤0.25	0.5	1	2	4	≥8			
LFX	‐	‐	107	66	16	35	19	18	0.25–8	1	27.6 (72/261)
MFX	7	47	112	21	20	26	19	9	0.06–8	0.25	36.4 (95/261)

*Note*: ‐, This concentration was not tested for the drug.

Abbreviations: LFX, levofloxacin; MFX, moxifloxacin; MIC, minimum inhibitory concentration; RR‐TB, rifampicin‐resistant tuberculosis.

**FIGURE 3:**
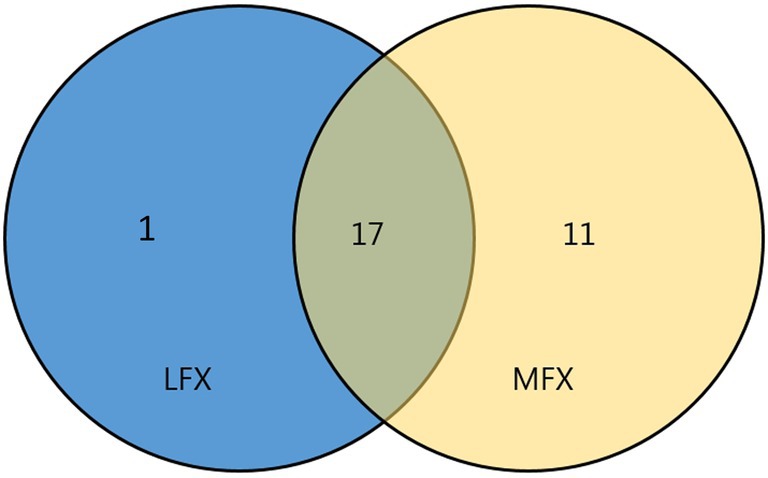
Strains with high‐level resistance (HLR) to levofloxacin (LFX) and moxifloxacin (MFX).

When using 4 μg/mL as the critical concentration for high‐level resistance (HLR) to LFX, the HLR rate for LFX was 25.0% (18/72). Using 2 μg/mL as the critical concentration for HLR to MFX, 29.5% (28/95) of MFX‐resistant strains exhibited HLR. In total, 17 strains were highly resistant to both LFX and MFX. Among 18 strains with HLR to LFX, the MIC of MFX for one strain was 2 μg/mL. Among the 28 strains with HLR to MFX, 11 strains were not highly resistant to LFX, including 2 strains with an MIC of 2 μg/mL and 9 strains with an MIC of 4 μg/mL (Figure 3).

### 3.4 Cross‐resistance to LFX and MFX

**FIGURE 4:**
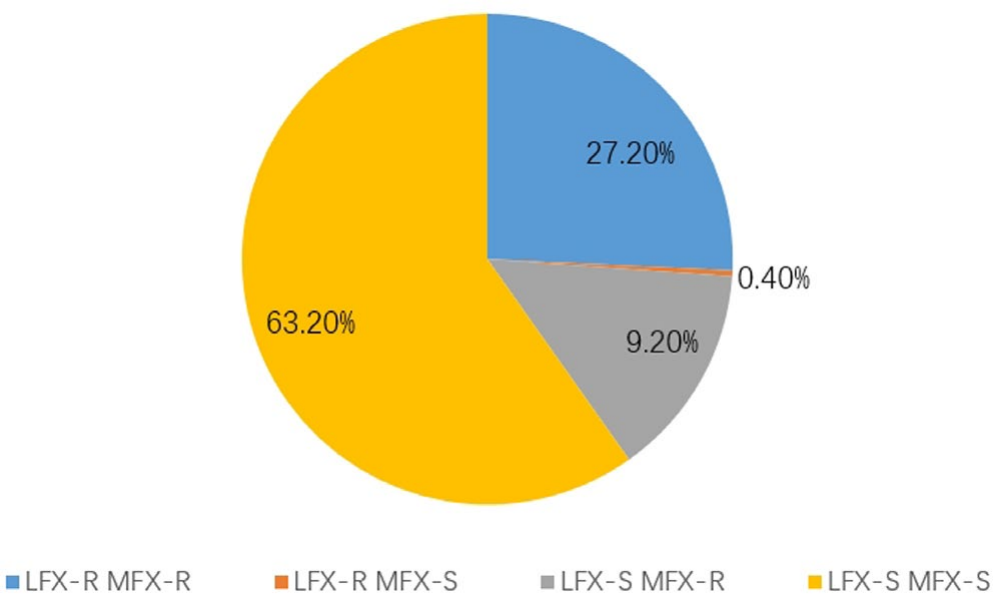
Cross‐resistance to levofloxacin (LFX) and moxifloxacin (MFX).

Among the 261 strains of RR‐TB, 27.2% (71/261) were resistant to both LFX and MFX, 0.4% (1/261) were resistant to LFX but sensitive to MFX, 9.2% (24/261) were sensitive to LFX but resistant to MFX, and 63.2% (165/261) were sensitive to both LFX and MFX (Figure 4).

### 3.5 MDR‐TB and pre‐XDR‐TB detection rates and trends

**FIGURE 5:**
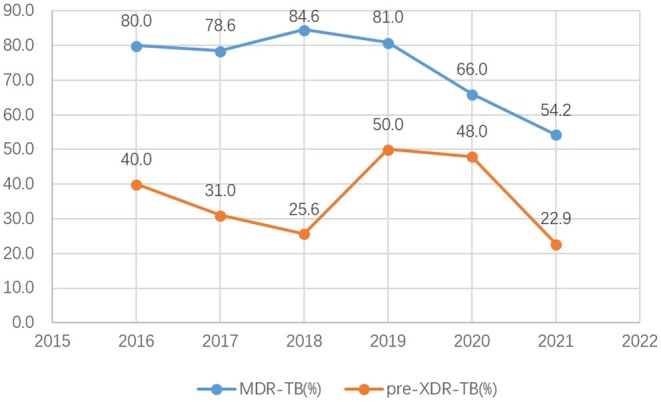
Trends of MDR‐TB and pre‐XDR‐TBdetection rates among patients with RR‐TB.

**FIGURE 6:**
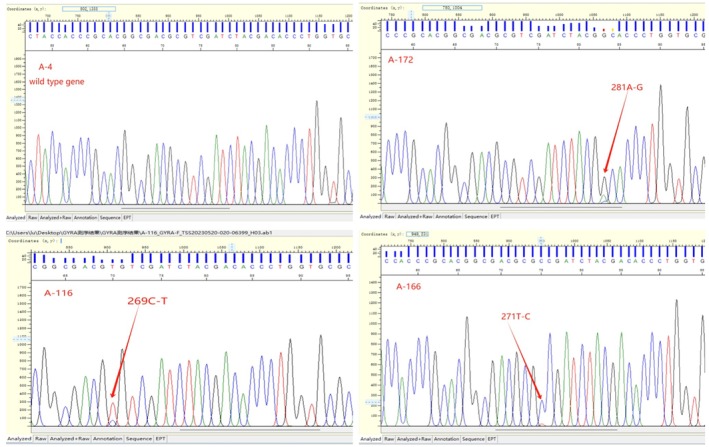
Sequencing map.:A‐4:,A wild type strain, no mutant site was detected. A‐172:,Mutation at site 281A‐G. A‐116, Mutation at site 269C‐T. A‐166,Mutation at site 271 T‐C.

According to the results of the microplate DSTs for LFX and MFX, the detection rates of MDR‐TB and pre‐XDR‐TB from 2016 to 2021 in patients with RR‐TB in the Beijing TB prevention system were 73.2% (191/261) and 36.4% (95/261), respectively. The MDR‐TB detection rate over this period significantly declined after rebounding in 2018, resulting in a statistically significant trend of change (*χ* ^2^ trend = 9.995, *p* = 0.002). The pre‐XDR‐TB detection rate slowly decreased from 2016 to 2018 and then continued to decline after a significant increase in 2019. By 2021, the rate had recovered to the 2018 level, and the trend of change was significant (*χ* ^2^ trend = 12.744, *p* = 0.026; Figure 5; Table 3). [Corrections added on 10 April 2025 after first online publication: The figure 4 has been revised and the citation of figures 5 and 6 have been updated in the article].

**TABLE 3:** Analysis of the MDR‐TB and pre‐XDR‐TB detection status and trends change over time.

Year	RR‐TB	MDR‐TB	pre‐XDR‐TB
2016	40	32	16
2017	42	33	13
2018	39	33	10
2019	42	34	21
2020	50	33	24
2021	48	26	11
*χ* ^2^ trend	–	9.995	12.744
p	–	0.002	0.026

### 3.6 Analysis of treatment outcomes and plans

Among the 261 patients with RR‐TB, 31 patients were excluded from the evaluation, and the remaining 230 patients underwent treatment. Among them, 107 patients were cured, and 60 completed the course of treatment, giving a treatment success rate of 72.6% (167/230). Treatment failed in 7 patients, 7 patients had adverse reactions during treatment, 8 patients died, and 40 patients had a treatment course exceeding 2 years. Based on these data, the treatment failure rate was 26.9% (62/230).

Among the 75 searchable medication regimens, the success rates for 2 regimens containing FQs were 82.6% (19/23) and 70.0% (21/30), with no difference noted (*χ* ^2^ = 1.1118, *p* = 0.290). Among the four LFX‐resistant patients, treatment failed in three patients, and the treatment outcome significantly differed according to LFX resistance (Fisher's exact test, *p* = 0.009). Meanwhile, treatment outcomes did not significantly differ based on MFX resistance (*χ* ^2^ = 1.213, *p* = 0.419; Table 4).

**TABLE 4:** Analysis of fluoroquinolones (FQs) sensitivity results and treatment outcomes in 75 patients with rifampicin‐resistant tuberculosis (RR‐TB).

Medication regimen (*n* = 75)	Success rate	Drug resistance situation	Treatment outcome			
			Treatment success	Treatment failure	*χ* ^2^	*p*
Including LFX (*n* = 23)	82.6% (19/23)	LFX resistance	1	3	—	0.009
		LFX sensitive	18	1		
Including MFX (*n* = 30)	70.0% (21/30)	MFX resistance	9	2	1.213	0.419
		MFX sensitive	12	7		

*Note*: ‐, *T* < 1 as analyzed using Fisher's exact test.

Abbreviations: LFX, levofloxacin; MFX, moxifloxacin.

### 3.7 Sequencing outcome of gyrA and gyrB

The distribution of various mutation types is summarized in Table 5. Among the total RR‐TB strains, mutations in the gyrA gene were detected in 28.0% (73/261) of isolates, whereas no mutations were found in the gyrB gene. Of the 73 mutant strains, 72 strains (98.6%, 72/73) exhibited single‐point mutations in the gyrA gene at sites 94, 90, 91, and 88. Specifically, 39 strains (53.4%, 39/73) had mutations at site 94, predominantly Asp94Ala and Asp94Gly, followed by 26 strains (35.6%, 26/73) with mutations at site 90, where Ala90Val was the most common mutation. Only one strain exhibited a double‐site mutation, which was a synonymous mutation. Some common mutation sites are shown in figure 6.

**TABLE 5:** Fluoroquinolones (FQs)‐resistant genes and distribution of different mutations.

Genes	Locus	Nucleotide change	Mutation type	No. of FQ‐resistant isolates with different mutations (*n* = 261)	Final confidence grading
gyrA				73 (28.0%)	
	88			2	
		262G‐T	Gly88Cys	2	(1) Assoc w R
	90			26	
		269C‐G	Ala90Gly	1	(4) Not assoc. w R ‐ Interim
		268G‐T	Ala90Ser	1	—
		269C‐T	Ala90Val	24	(1) Assoc w R
	91			5	
		271 T‐C	Ser91Pro	5	(1) Assoc w R
	94			39	
		281A‐C	Asp94Ala	23	(1) Assoc w R
		280G‐A	Asp94Asn	1	(1) Assoc w R
		281A‐G	Asp94Gly	14	(1) Assoc w R
		280G‐T	Asp94Tyr	1	(1) Assoc w R
	88and91			1	
		264C‐T, 273G‐T	Gly88Gly, Ser91Ser	1	Synonymous mutation
gyrB				0	
No mutation				188 (72.0%)	

*Note*: ‐,No credible evidence linking mutations to drug resistance was found.

## 4 DISCUSSION

FQs can bind to bacterial DNA gyrase to prevent DNA replication and transcription, leading to DNA degradation and bacterial death, thereby achieving the goal of killing MTB. These drugs can also enter macrophages and synergize with other drugs to enhance anti‐TB effects.^11^ Anti‐TB treatment regimens containing FQs can improve the rate of sputum negativity, thus reducing the infection period, which is of great significance for controlling the source of infection and reducing the spread of TB.^12^ After WHO redefined pre‐XDR‐TB and updated the treatment plan for DR‐TB, only 50% of patients with RR‐TB registered globally underwent DST for FQs in 2021 despite concerns about FQs resistance.^13^ In China, FQs are widely used. In 2017, FQs ranked first in terms of consumption and intensity of use. For many years, LFX and MFX were the most commonly used agents among all antibacterial drug varieties.^14^ Beijing is the economic and political center of China, and it has a large floating population and a high level of antibiotic use.^15^ FQs face greater risks of drug resistance. According to the existing drug resistance screening policy in Beijing, only the traditional proportional method of LFX DST is conducted for patients with RR‐TB, and MFX DST is not routinely performed. In terms of feedback times and drug types, the existing screening policy can no longer meet the needs of clinical diagnosis and treatment, and this situation seriously affects the formulation of Beijing's DR‐TB prevention and control strategies.

This study investigated 8512 clinical isolates cultured from patients with pulmonary TB who visited Beijing TB prevention system and designated hospitals between 2016 and 2021. Of these, 573 duplicate strains were excluded. Among the 7939 remaining strains, 1232 strains were selected from high‐risk patients for drug resistance screening. Then, 261 strains of RR‐TB bacteria were selected using the traditional solid proportional DST, and the resistance to LFX and MFX, two core drugs in Group A, was detected using the microplate method. The cohort of patients with resistance to LFX and MFX had a male predominance, and most patients were aged between 20 and 59 years. Most patients were newly treated. The rates of resistance and sensitivity to LFX and MFX differed in terms of age and treatment history. Compared to the sensitive group, the proportions of patients older than 60 years and retreated patients were higher in the resistant group, which is consistent with the 2007 national drug resistance monitoring results of the China Center for Disease Control and Prevention,^16^ indicating that elderly people and retreated patients are more prone to FQ resistance.

The World Tuberculosis Report released by WHO in 2023 mentioned that in 2022, pre‐XDR‐TB accounted for 18% of global MDR/RR‐TB cases.^1^ The current study found that among patients diagnosed with RR‐TB from 2016 to 2021, resistance rates for LFX and MFX were 27.6% and 36.4%, respectively, slightly exceeding the proportion of patients with MDR/RR‐TB in China who were resistant to FQs in 2019 (27%).^17^ Some studies reported that the MFX resistance rate ranges from 17% to 62% in various countries.^18^, ^19^, ^20^ Yin et al.^21^ conducted a retrospective study of patients with MDR‐TB in Beijing from 2018 to 2020 using whole‐genome sequencing to predict drug resistance. They found that the FQ resistance rate in patients with newly diagnosed MDR‐TB was as high as 39.7%. FQ resistance monitoring in patients with MDR‐TB in southwestern China from 2014 to 2016 revealed that resistance rates for LFX and MFX were 50% and 38.6%, respectively.^22^ In southern China, among 400 patients resistant to any anti‐TB drug at Guangzhou Chest Hospital, the resistance rates for LFX and MFX were as high as 75.75% and 73%, respectively.^23^ Although there are differences in FQ resistance rates in different regions and populations related to the genotype, detection method, strain source, sample size, and other factors, the findings suggest that the situation of FQ resistance among patients with TB in China is not optimistic, and the problem of FQ resistance cannot be ignored.

Wang et al.^24^ reported that among 130 strains resistant to ofloxacin, 110 strains were resistant to LFX, and 108 strains were resistant to MFX. In addition, 90.9% (100/110) of LFX‐resistant strains were resistant to MFX, indicating a high level of cross‐resistance. Among the 261 strains of RR‐TB investigated in this study, 71 were simultaneously resistant to both LFX and MFX. One LFX‐resistant strain was sensitive to MFX, whereas 24 LFX‐sensitive strains were resistant to MFX, indicating incomplete bidirectional cross‐resistance.^9^ WHO proposed an alternative DST solution for cross‐resistant strains in the “optimized broth microdilution plate methodology for drug susceptibility testing of MTB complex” and suggested using the LFX clinical breakpoint of 4 μg/mL as the surrogate for the MFX clinical breakpoint of 2 μg/mL.^4^ In this study, using 2 μg/mL as the critical concentration of HLR to MFX, we detected 28 strains with high‐level MFX resistance. Using 4 μg/mL as the critical concentration of HLR to LFX, 18 strains were highly resistant to LFX, including 17 strains (94.4%,17/18) with HLR to MFX. 11 (39.3%,11/28) strains with HLR to MFX were misidentified as having low tolerance. Therefore, more experimental data are needed using MFX HLR detection in place of LFX HLR detection.

Xia et al.^25^ analyzed strains undergoing national drug resistance monitoring and found that from 2007 to 2013, the MFX resistance rate among patients with MDR‐TB increased from 24.4% to 56.4%. In a study of the entire TB population, the LFX resistance rate among clinical isolates in Beijing Chest Hospital increased from 15.4% in 2005 to 25.7% in 2015, and the HLR rate increased from 16.7% to 40.6% over the same period.^26^ Our study found that the rates of MDR‐TB and pre‐XDR‐TB among patients with RR‐TB displayed an overall downward trend during 2016–2021, and the detection trends of both types of TB were statistically significant. On the one hand, these findings are related to the adjustment of the drug resistance screening policy in Beijing. Since September 2018, the city's TB prevention and control institutions and designated hospitals have conducted rifampin resistance screening for all patients with newly diagnosed TB in accordance with the “Drug Resistance Screening Process” in Beijing, which can timely detect RR‐TB, adjust the treatment plan, and reduce the occurrence of DR‐TB. Meanwhile, the use of GeneXpert testing between 2019 and 2021 permitted more accurate detection of RR‐TB. On the other hand, under the influence of the COVID‐19 epidemic environment, delayed diagnosis and discovery of DR‐TB might have resulted in a decrease in the detection rate. In the past 10 years, the extensive application of FQs in clinical practice, especially in the field of respiration and TB in China, has led to an increasing drug resistance rate over time, representing a problem that cannot be ignored in TB control. As the capital of China, Beijing should continue to strengthen the trend of FQs resistance in patients with pulmonary TB, especially RR‐TB.

The treatment success rate for patients with RR‐TB in this study was 72.6% (167/230), exceeding those of 57% and 54% for patients with MDR/RR‐TB registered globally and in China, respectively, in 2017.^17^ The success rates of LFX‐ and MFX‐containing regimens were 82.6% and 70.0%, respectively, which were not significantly different. 4 patients received LFX despite carrying LFX‐resistant strains, and treatment failed in 3 patients. Treatment outcomes significantly differed between patients with and without LFX resistance. Therefore, timely adjustment of treatment regimen should be performed based on the results of drug resistance testing to improve the success of treatment. 11 patients who were resistant to MFX still received the drug, and treatment failed in 2 patients. The treatment outcomes did not differ based on MFX resistance. There are several possible reasons for this finding. First, other drugs in the combination regimens might have exerted anti‐TB effects. Second, mutual synergistic effects occurred among the drugs. Finally, this study referred to WHO standards, and the critical concentration of MFX resistance is 0.25 μg/mL. Some reports stated that 1 μg/mL should be the breakpoint for determining MFX resistance. Different judgment criteria result in differences in statistical results.^27^ However, as MFX is a broad‐spectrum antibiotic that can be used for the treatment of various infections, the treatment regimen should be adjusted based on the results of drug resistance testing to prevent the development of drug resistance.

DNA gyrase mutations were found to be associated with resistance to FQs. The gyrA gene mutation can explain 50%–95% of the clinical isolates' resistance phenotype to FQs, whereas the gyrB gene mutation can explain 0%–30%, and the prevalence of gene mutations vary in different regions.^28^, ^29^, ^30^, ^31^ In this study, we identified gyrA gene mutations in 28.0% (73/261) of the isolates, whereas no mutations in gyrB gene were found. Notably, 27.6% (72/261) of RR‐TB strains exhibited mutations at a single locus. The common mutation sites were 94 (53.4%, 39/73), 90 (35.6%, 26/73), 91 (6.8%, 5/73), and 88 (2.7%, 2/73), respectively. Previous research has indicated that the Asp94Ala and Ala90Val mutations are the predominant mutations conferring resistance to FQs,^32^, ^33^ and these findings are consistent with the results of our study. According to the WHO's Catalogue of Mutations in the MTB complex and their association with drug resistance (Catalogue),^34^, ^35^ 70 mutant strains (95.9%,70/73) have been classified as group 1: Assoc w R, which suggested the mutations having a strong correlation with drug resistance. The Ala90Gly mutation is classified as group 4: Not assoc. w R‐Interim, which is moderately related to non‐drug resistance. However, the phenotypic drug sensitivity observed in this study suggested a low‐level drug of resistance for this strain (LFX: 2 μg/mL and MFX: 0.5 μg/mL). This indicated that certain mutations may only cause a minor increase in MIC values. Further data on mutations at this site are required to elucidate the relationship between these mutations and drug resistance. A novel mutation Ala90Ser was discovered, and no correlation with drug resistance was indicated in the catalogue. The phenotypic drug sensitivity observed for this isolate suggested sensitive, implying that this mutation may not contribute to drug resistance. However, as only one strain exhibiting this mutation was detected, its correlation with drug resistance still needs further data confirmation.

Regarding the study limitations, this research selected patients with RR‐TB screened by the Beijing TB prevention system and designated hospitals, and thus, cases in non‐designated hospitals could have been missed, biasing the results. This study only sequenced the gene of gyrA and gyrB, and the molecular mechanisms of drug resistance and the reasons for cross‐resistance were not thoroughly investigated.

In conclusion, this is the first report that highlights the rising prevalence of FQs resistance in RR‐TB patients in Beijing. Both phenotypic and sequencing resistance tests indicate a critical level of FQs resistance. The strains exhibited partial cross‐resistance between LFX and MFX. It is recommended to test for FQs resistance and develop appropriate treatment strategies. Additionally, attention should be paid to evolving trends in MDR‐TB and pre‐XDR‐TB. Early detection for drug‐resistant, followed by prompt initiation of standardized treatment, is essential to reduce the spread of DR‐TB.

## AUTHOR CONTRIBUTIONS

**Wang Nenhan:** Methodology; writing – original draft. **Tian Lili:** Conceptualization; data curation. **Zhao Yanfeng:** Resources; software. **Chen Shuangshuang:** Resources; software. **Tao LiYing:** Formal analysis. **Li Qiao:** Investigation; resources. **Li Chuanyou:** Methodology; project administration; writing – review and editing. **Dai Xiaowei:** Funding acquisition; methodology; project administration; writing – review and editing.

## FUNDING INFORMATION

The present work was supported by the Research and Cultivation Special Project of Beijing Center for Disease Prevention and Control (2023‐KYJH‐03).

## CONFLICT OF INTEREST STATEMENT

The authors report no conflicts of interest.

## ETHICS STATEMENT

This study was approved by the Institutional Ethics Review Committee (approval number: 2023 no. [13]).

## References

[1] World Health Organization . Global Tuberculosis Report 2023. World Health Organization; 2023.

[2] WHO . WHO Consolidated Guidelines on Tuberculosis. Module 4: Treatment‐Drug‐Resistant Tuberculosis Treatment (2020). 32603040

[3] Technical Report on Critical Concentrations for Drug Susceptibility Testing of Medicines Used in the Treatment of Drug‐Resistant Tuberculosis. World Health Organization; 2018.

[4] World Health Organization . Optimized Broth Microdilution Plate Methodology for Drug Susceptibility Testing of Mycobacterium Tuberculosis Complex. World Health Organization; 2022.

[5] Guoping L , Xinran L . Research on the influence of coordinated development strategy of Beijing‐Tianjin‐Hebei on Beijing's population scale control. J Hebei Univ Econ Bus. 2021;42(3):94‐102.

[6] Technical Manual for Drug Susceptibility Testing of Medicines Used in the Treatment of Tuberculosis. Licence: CC BY‐NC‐SA 3.0 IGO.

[7] CLSI . Performance standards for susceptibility testing of mycobacteria, *Nocardia* spp., and other aerobic Actinomycetes. CLSI supplement M24S. 2nd ed. Clinical and Laboratory Standards Institute; 2023. 31339680

[8] World Health Organization . Meeting Report of the WHO Expert Consultation on the Definition of Extensively Drug‐Resistant Tuberculosis. World Health Organization; 2021.

[9] Chinese Antituberculosis Association . Guidelines for chemotherapy of drug resistant tuberculosis. Chin J Antituberc. 2019;41(10):1025‐1073. doi:10.3969/j.issn.1000-6621.2019.10.001

[10] Zhao YL , Chen MT . Technical Guidelines for Tuberculosis Prevention and Control in China. Beijing: People's Medical Publishing House; 2021:183‐186.

[11] Chen TC , Lu PL , Lin CY , Lin WR , Chen YH . Fluoroquinolones are associated with delayed treatment and resistance in tuberculosis: a systematic review and meta‐analysis. Int J InfectDis. 2011;15(3):e211‐e216. 10.1016/j.ijid.2010.11.008 21195001

[12] Shenjie T . Re evaluation of the application of fluoroquinolones in drug‐resistant tuberculosis. Chinese J Tuber Respir Dis. 2014;37(10):727‐729. 25537404

[13] World Health Organization . Global Tuberculosis Report 2021. World Health Organization; 2021.

[14] National Health Commission of the People's Republic of China . Report on the Management and Resistance Status of Antibiotics in China (2018). Peking Union Medical College Press; 2019.

[15] Wushouer H , Zhou Y , Zhang X , et al. Secular trend analysis of antibiotic utilisation in China's hospitals 2011‐2018, a retrospective analysis of procurement data. Antimicrob Resist Infect Control. 2020;9(1):53. doi:10.1186/s13756-020-00709-6 32295639 PMC7160954

[16] Zhao Y , Xu S , Wang L , et al. National Survey of drug‐resistant tuberculosis in China. N Engl J Med. 2012;366:2161‐2170. 22670902 10.1056/NEJMoa1108789

[17] Caihong X , Yanlin Z . To evaluate the tuberculosis prevention and control situation in China from the perspective of global tuberculosis report 2020. Chin J Infect Dis. 2021;39(7):392‐397. doi:10.3760/cma.j.cn311365-20210406-00117

[18] Mamatha HG , Shanthi V . Baseline resistance and cross‐resistance among fluoroquinolones in multidrug‐resistant *Mycobacterium tuberculosis* isolates at a national reference laboratory in India. J Glob Antimicrob Resist. 2018;12:5‐10. doi:10.1016/j.jgar.2017.08.014 28887289

[19] Chuang PH , Wu MH , Fan SY , Lin KY , Jou R . Population‐based drug resistance surveillance of multidrug‐resistant tuberculosis in Taiwan, 2007‐2014. PLoS One. 2016;11(11):e0165222. doi:10.1371/journal.pone.0165222 27846235 PMC5112772

[20] Zignol M , Dean AS , Alikhanova N , et al. Population‐based resistance of *Mycobacterium tuberculosis* isolates to pyrazinamide and fluoroquinolones: results from a multicountry surveillance project. Lancet Infect Dis. 2016;16(10):1185‐1192. doi:10.1016/S1473-3099(16)30190-6 27397590 PMC5030278

[21] Yin J , Zhang H , Gao Z , et al. Transmission of multidrug‐resistant tuberculosis in Beijing, China: an epidemiological and genomic analysis. Front Public Health. 2022;10:1019198. doi:10.3389/fpubh.2022.1019198 36408017 PMC9672842

[22] Zheng H , He W , Jiao W , et al. Molecular characterization of multidrug‐resistant tuberculosis against levofloxacin, moxifloxacin, bedaquiline, linezolid, clofazimine, and delamanid in southwest of China. BMC Infect Dis. 2021;21(1):330. 33832459 10.1186/s12879-021-06024-8 PMC8028109

[23] Hameed HMA , Tan Y , Islam MM , et al. Phenotypic and genotypic characterization of levofloxacin‐ and moxifloxacin‐resistant *Mycobacterium tuberculosis* clinical isolates in southern China. J Thorac Dis. 2019;11(11):4613‐4625. 31903250 10.21037/jtd.2019.11.03 PMC6940237

[24] Wang Q , Song Y , Pang Y , et al. Study on the cross‐resistance among 5 different fluroquinolones in ofloxacin‐resistant *Mycobacterium tuberculosis* isolates. Chin J Antituberc. 2014;36(6):453‐457. doi:10.3969/j.issn.1000-6621.2014.06.009

[25] Xia H , Zheng Y , Liu D , et al. Strong increase in Moxifloxacin ResistancWe rate among multidrug‐resistant *Mycobacterium tuberculosis* isolates in China, 2007 to 2013. Microbiol Spectr. 2021;9(3):e0040921. doi:10.1128/Spectrum.00409-21 34851179 PMC8635133

[26] Huo F , Zhang F , Xue Y , et al. Increased prevalence of levofloxacin‐resistant *Mycobacterium tuberculosis* in China is associated with specific mutations within the gyrA gene. Int J Infect Dis. 2020;92:241‐246. 31978580 10.1016/j.ijid.2020.01.021

[27] Ou X , Song Z , Zhao B , et al. Diagnostic efficacy of an optimized nucleotide MALDI‐TOF‐MS assay for anti‐tuberculosis drug resistance detection. Eur J Clin Microbiol Infect Dis. 2024;43(1):105‐114. doi:10.1007/s10096-023-04700-y 37980301

[28] Zhang Z , Lu J , Wang Y , Pang Y , Zhao Y . Prevalence and molecular characterization of fluoroquinolone‐resistant *Mycobacterium tuberculosis* isolates in China. Antimicrob Agents Chemother. 2014;58(1):364‐369. doi:10.1128/AAC.01228-13 24165186 PMC3910797

[29] Mokrousov I , Otten T , Manicheva O , et al. Molecular characterization of ofloxacin‐resistant *Mycobacterium tuberculosis* strains from Russia. Antimicrob Agents Chemother. 2008;52(8):2937‐2939. doi:10.1128/AAC.00036-08 18559646 PMC2493099

[30] Sirous M , Khosravi AD , Tabandeh MR , Salmanzadeh S , Ahmadkhosravi N , Amini S . Molecular detection of rifampin, isoniazid, and ofloxacin resistance in Iranian isolates of *Mycobacterium tuberculosis* by high‐resolution melting analysis. Infect Drug Resist. 2018;11:1819‐1829. doi:10.2147/IDR.S178831 30425535 PMC6202043

[31] Li M , Zhang Y , Wu Z , et al. Transmission of fluoroquinolones resistance among multidrug‐resistant tuberculosis in Shanghai, China: a retrospective population‐based genomic epidemiology study. Emerg Microbes Infect. 2024;13(1):2302837. doi:10.1080/22221751.2024.2302837 38205528 PMC10810664

[32] Zhang ZG , Du CY , Zhang Q , et al. Analysis of the relationship between gyrA mutations and fluoroquinolone levels in *Mycobacterium tuberculosis* isolates from China. Chinese J Antitubercul. 2016;38(9):706‐711.

[33] Miotto P , Tessema B , Tagliani E , et al. A standardised method for interpreting the association between mutations and phenotypic drug resistance in *Mycobacterium tuberculosis* . Eur Respir J. 2017;50:1701354. doi:10.1183/13993003.01354-2017 29284687 PMC5898944

[34] World Health Organization . Catalogue of Mutations in Mycobacterium tuberculosis Complex and their Association with Drug Resistance. World Health Organization; 2021.

[35] World Health Organization . Catalogue of Mutations in Mycobacterium tuberculosis Complex and their Association with Drug Resistance. 2nd ed. World Health Organization; 2023.

